# Comparison of the Effects of Spreader Graft and Overlapping Lateral Crural Technique on Rhinoplasty by Rhinomanometry

**Published:** 2013-06

**Authors:** Mahmoud Omranifard, Hosein Abdali, Mehdi Rasti Ardakani, Amiryousef Ahmadnia

**Affiliations:** Department of Plastic Surgery, School of Medicine, Isfahan University of Medical Sciences, Isfahan, Iran

**Keywords:** Spreader graft, Lateral crural technique, Rhinoplasty, Rhinomanometry

## Abstract

**BACKGROUND:**

Nasal valve collapse and especially internal nasal valve insufficiency is a common cause of nasal airway obstruction. This study compares the effects of spreader graft and overlapping lateral crural technique on rhinoplasty by rhinomanometry.

**METHODS:**

Fifty patients were randomly assigned into two groups and underwent spreader graft or overlapping lateral crural technique. Objective assessment was performed by clinical examination and rhinomanometry before and after rhinoplasty.

**RESULTS:**

Nasal obstruction had no significant difference before and after rhinoplasty and no significant difference was observed between surgical techniques. Right, left and total nasal flow and resistance were different before and after surgery but were not significant. Base of the nose was not significantly different between two groups, but nasal projection was 2 mm in the the group who underwent overlapping lateral crura technique and the difference was statistically significant. Our study showed that both overlapping lateral crura and spreader graft technique were beneficial in rhinoplasty and they could provide enough internal nasal valve support. The overlapping lateral crura was an appropriate surgical technique for tip projection in comparison to spreader graft.

**CONCLUSION:**

The overlapping lateral crura technique was shown to be a better surgical way for tip projection in comparison to spreader graft.

## INTRODUCTION

Breath is a life performance that results into oxygenation of the body tissues. When breathing through the nose is diverted from its normal route, side effects would affect all body tissues. Different nasal respiratory problems may be caused by surgery including maintaining a deviated septum, enlarged nasal prongs, nasal allergies, nasal valve stenosis and narrowing of the nostril.^1^^-^^3^ Valve area is one of the most complicated and sensitive part of the nasal airway, which includes the area between the caudal septum, the caudal superolateral cartilage, anteroinferior part of inferior turbinate and the surrounding tissue.^4^

Nasal valve collapse and especially internal nasal valve insufficiency is a common cause of nasal airway obstruction. The internal nasal valve stenosis can be congenital or caused by trauma or complications of previous surgery or dropping nasal tip.^5^^-^^7^ Weakness in the superolateral cartilage and narrowing the valve can cause stricture and blockage of the nasal passages, collapse of the lateral nasal wall and exacerbate asthma, especially in patients at increased during breathing through the nose.^8^^,^^9^ In evaluating dyspnea, different methods are used. One of these methods is nowadays considered approach of rhinomanometry that the air flow is resistant and is simultaneously measured.^10^^-^^12^

Today, the most common technique used for structural reform under the nose is rhinoplasty.^13^ Although many techniques have been devised to correct both the functional and aesthetic aspects of this problem, none is uniformly successful. One of the most common techniques used in structural reform is spreader graft and the other technique used in the field is overlapping lateral crura.^14^^-^^16^ In all these techniques the patient’s postoperative function improvement is important.^17^ Techniques can be compared with each other by subjective methods as questionnaire or using objective (paraclinical) methods such as rhinomanometry.

Most of studies for this technique are based on clinical examination and change of postoperative complaints and are not very precise to reveal the advantage of one technique over another. Therefore, recent studies have focused on clinical aspects such as rhinomanometry to compare the surgical techniques. In this study, we compared the common complaints of the patient’s rate of recovery with one objective method (rhinomanometry). This study compares the effects of spreader graft and overlapping lateral crural technique on rhinoplasty by rhinomanometry.

## MATERIALS AND METHODS

The present study is a clinical trial on patients for elective surgery of nose (rhinoplasty) with two techniques of spreader graft and overlapping lateral crural. The study was undertaken in Al-Zahra Hospital. Rhinomanometry was done in two stages before and three months after surgery. We included patients with no clinical problems and individual consent to participate in research projects. Exclusion criteria were structural problems such as nasal obstruction, a brief history of the deformity requiring rhinoplasty deviated septum, hardening surgery, an unwillingness to continue participation in the plan, new clinical conditions or a change in therapeutic techniques and lack of access to patients referred for evaluation of a person after the operation. 

Rhinomanometry was done with the Recommended International Standards Committee and was performed in a facility by a technician. After rhinomanometry, patients were randomly divided into two groups and surgeries were performed by a group of surgeons. One group of patients with speader graft and others were operated by overlapping lateral crura technique. Patients were monitored for a month after surgery and then again after three months under the same initial conditions undertaken by rhinomanometry. In two stages of rhinomanometry, nasal resistance was evaluated by Pascal per milliliter per second (Pa/mL/s). The right and left and total nasal flow index were evaluated in two groups by milliliter per second (mL/s). 

In two stages of rhinomanometry, the clinical complaints of patients were assessed by the standard Visual Analogue Scale (VAS). Anthropometric characteristics of patients (base and tip projection of the nose) were measured by taking photo and computer imaging technique preoperative and postoperative. Finally, the data were analyzed by the SPSS software (Version 18, Chicago, IL, USA). Data were expressed as mean±SD. Dependent T test and ANOVA were used for data analysis. The significance level was considered as p <0.05. Ethical principles of the Declaration of Helsinki have been under consideration.

## RESULTS

In this study, 50 patients were enrolled. In speader graft group, 19 men (76%) and 6 women (24%) and in lateral crural overlapping group, 17 men (68%) and 8 women (32%) participated. Mean age of patients in speader graft group was 35.6±14.9 and in overlapping lateral crural group was 37.1±17.1 years. 

Before rhinoplasty, based on a medical examination and rhinomanometry findings, 3 patients (6%) had mild obstruction and 47 patients (94%) had no obstruction. After speader graft technique, 2 patients (8%) had mild obstruction and 23 patients (92%) had no complaint. Following overlapping lateral crural technique, 1 patient (4%) had mild obstruction and 24 patients (96%) had no complaint. Statistically, based on medical examination and rhinomanometry findings, nasal obstruction had no significant difference before and after rhinoplasty and no significant difference was observed between surgical techniques (P>0.05).

Before rhinoplasty, based on clinical complaints of obstruction, 1 patient (2%) had mild obstruction and 49 patients (98%) had no complaints. After speader graft technique, 1 patient (4%) had mild obstruction and 24 patients (96%) had no complaint. Following overlapping lateral crural technique, 1 patient (4%) had mild obstruction and 24 patients (96%) had no complaint. Statistically based on clinical complaints, nasal obstruction had no significant difference before and after rhinoplasty and no significant difference was observed between surgical techniques (P>0.05). Rhinomanometry results before and after surgeries are shown in Table 1.

**Table 1:** Rhinomanometry results before and after two methods of surgeries

**Mean±SD after lateral crural over lapping (postoperative)**	**Mean±SD after speader graft** **(postoperative)**	**Mean±SD preoperative**	**Rhinomanometric index**
340±129.34	345.86±134.63	350.60±133.17	Right nasal flow
399.34±192.19	405.53±181.01	411.22±141.17	Left nasal flow
739.94±260.50	751.39±255.09	761.82±267.87	Total nasal flow
0.51±0.33	0.45±0.24	0.40±0.48	Right nasal resistance
0.57±0.39	0.49±0.40	0.43±0.66	Left nasal resistance
0.22±0.16	0.20±0.14	0.19±0.24	Total nasal resistance

(Right, left and total nasal flow based on mL/s and right and left and total nasal resistance based on Pa/mL/s).

Right and left and total nasal flow and right and left and total resistance before and after surgery were difference but not significant (P>0.05). However, no significant difference was observed between two techniques (P>0.05). 

The anthropometric characteristics of the patients included the base of the nose and nasal projection. Base of the nose was not significantly different between 2 groups but nasal projection was 2 mm in the group who underwent overlapping lateral crural technique and when compared with spreader graft technique was statistically significant (P>0.05).

## DISCUSSION

The results of this study showed no significant difference in nasal obstruction before and after surgery in each group and between two groups. As we enrolled patients with no clinical problems, obstruction and rhinomanometry did not change significantly between groups but rhinomanometry findings decreased after surgery.

Rhinomanometric indexes decreased 3 months after surgery, because inflammation was observed in clinical examination at the site of surgery. The important problem was internal nasal valve insufficiency and obstruction after rhinoplasty. So using techniques to reshape and add structure to the lateral crural to create the desired lateral crural contour can provide sufficient alar rim and internal nasal valve support.

 In Okhovat *et al.* study, rhinomanometry was performed on 48 patients before and after septoplasty. The findings indicated that in addition to reduction in symptoms of obstruction, rhinomanometry showed an increase in flow rate and the total flow on both sides of the nose and a reduction in resistance to objective results. It can be recommended as a way to evaluate the results of septoplasty and to predict structural reforms.^18^ Also Jassen and colleagues evaluated the effect of rhinomanometry on 92 patients undergoing rhinoplasty. Rhinomanometry findings revealed an improvement among 56 patients with midline deviation and in 36 patients without any septal deviation.^19^ Tombu’s study showed that the evaluation of rhinoseptoplasty results via rhinomanometry denoted to an effectively improved surgical techniques, although further studies are needed to evaluate the usefulness of these techniques.^20^ In another study, Tiukin showed that rhinomanometry technique can be compared with various surgical procedures in the practical use.^21^

There are more studies to compare techniques together such as spreader graft and overlapping lateral crural method. For example in one study, the efficacy of surgery of Apaydin lateral crural turn-in flap in relieving of nasal structural abnormalities in 24 patients showed that this technique could improve the performance of reform and opening internal lateral crural technique to be effective.^22^ Janis and colleagues in a retrospective study on 23 patients who underwent lateral crural turnover flap rhinoplasty procedure showed that this technique could be very effective on structural reforms and deformity including internal valve stenosis.^23^ Also, Boccieri *et al.* followed up 60 patients with structural abnormalities after speader graft procedure and showed that none of the patients complained from physiological disorders such as sparseness and self-induced internal valve dysfunction.^24^ In all these studies, there were no objective methods for patient examination. To compare existing techniques without using rhinomanometry and based on clinical signs and symptoms, overlapping lateral crural has been more effective in improving nasal tip although there are numerous studies showing that the spreader graft method was effective in improving the nasal structure and to prevent nasal valve collapse.^25^

Nasal tip reform greatly is dependent on the surgical technique. These studies on lapping lateral crural procedure were similar to our results. So in Wise and colleagues study, the projection improved in all patients.^26^ Also the modern approach to functional rhinoplasty considered the importance of the tip framework’s structural integrity. Sazgar *et al.* performed rhinoplasty on 5 patients by using overlapping lateral crural technique. This preliminary study showed that the hinged flap was an option in nasal tip reduction surgery that may provide an improvement in long-term aesthetic and functional outcomes through preservation of the nasal valve area.^27^

These results are similar to our study regarding tip deprojection. Their study showed that overlapping lateral crural technique improved functional outcomes through preservation of the nasal valve area. Our results could not support this finding because we did second rhinomanometry three months after rhinoplasty. However clinical examination showed inflammation in the site of surgery, so we needed more time for patient’s examination to get better results. Also Hossam *et al.* showed that in lateral crural overlay, there were an increase in tip rotation and a decrease in tip projection. They used computer imaging technique for assessment.

We included the patients for cosmetic rhinoplasty and suggested comparing of the technique on patients with clinical problems such as severe concavities and nasal obstruction by using dynamic objective methods for assessment in future studies. Due to inflammation at the site of surgery, our result was not significant in second assessment, so we suggested that patient evaluation must be done in longer periods. 

In conclusion, overlapping lateral crural and spreader graft were useful surgical techniques in rhinoplasty and they could provide enough support for internal nasal valve but overlapping lateral crural procedure was an appropriate surgical technique for tip deprojection. Use of other techniques with overlapping lateral crural may be more imperative. The results of the present study showed the effectiveness of both techniques for patients, but it is necessary to explain that each technique has specific application and can not be replaced by another and a plastic surgeon can select an effective technique for each patient according to aesthetic and therapeutic parameters. 

## CONFLICT OF INTEREST

The authors declare no conflict of interest.

## Footenote

Please cite this paper as:

Omranifard M, Abdali H, Rasti Ardakani M, Ahmadnia AY. Comparison of the Effects of Spreader Graft and Overlapping Lateral Crural Technique on Rhinoplasty by Rhinomanometry. World J Plast Surg 2013;2(2): 99-103.
